# Amphibian tolerance to arsenic: microbiome-mediated insights

**DOI:** 10.1038/s41598-024-60879-w

**Published:** 2024-05-03

**Authors:** Isabella Ferreira Cordeiro, Camila Gracyelle de Carvalho Lemes, Angélica Bianchini Sanchez, Ana Karla da Silva, Camila Henriques de Paula, Rosilene Cristina de Matos, Dilson Fagundes Ribeiro, Jéssica Pereira de Matos, Camila Carrião Machado Garcia, Marina Beirão, C. Guilherme Becker, Maria Rita Silvério Pires, Leandro Marcio Moreira

**Affiliations:** 1 Núcleo de Pesquisas em Ciências Biológicas, Universidade Federal de Ouro Preto, Ouro Preto, MG 35400-000 Brazil; 2 Laboratório de Genômica e Interação Bactérias-Ambiente, Departamento de Ciências Biológicas, Instituto de Ciências Exatas e Biológicas, Universidade Federal de Ouro Preto, Ouro Preto, MG 35400-000 Brazil; 3 Departamento de Biodiversidade Evolução e Meio Ambiente, Instituto de Ciências Biológicas, Universidade Federal de Ouro Preto, Belo Horizonte, MG 31270-901 Brazil; 4 Department of Biology, One Health Microbiome Center, Center for Infectious Disease Dynamics, Huck Institutes of the Life Sciences, Pennsylvania State University, University Park, PA 16802 USA

**Keywords:** Biotechnology, Ecology, Microbiology, Ecology, Environmental sciences

## Abstract

Amphibians are often recognized as bioindicators of healthy ecosystems. The persistence of amphibian populations in heavily contaminated environments provides an excellent opportunity to investigate rapid vertebrate adaptations to harmful contaminants. Using a combination of culture-based challenge assays and a skin permeability assay, we tested whether the skin-associated microbiota may confer adaptive tolerance to tropical amphibians in regions heavily contaminated with arsenic, thus supporting the adaptive microbiome principle and immune interactions of the amphibian mucus. At lower arsenic concentrations (1 and 5 mM As^3+^), we found a significantly higher number of bacterial isolates tolerant to arsenic from amphibians sampled at an arsenic contaminated region (TES) than from amphibians sampled at an arsenic free region (JN). Strikingly, none of the bacterial isolates from our arsenic free region tolerated high concentrations of arsenic. In our skin permeability experiment, where we tested whether a subset of arsenic-tolerant bacterial isolates could reduce skin permeability to arsenic, we found that isolates known to tolerate high concentrations of arsenic significantly reduced amphibian skin permeability to this metalloid. This pattern did not hold true for bacterial isolates with low arsenic tolerance. Our results describe a pattern of environmental selection of arsenic-tolerant skin bacteria capable of protecting amphibians from intoxication, which helps explain the persistence of amphibian populations in water bodies heavily contaminated with arsenic.

## Introduction

Arsenic (As) is one of the most abundant non-essential metalloids found in nature and it is highly toxic to living organisms across the food chain^1^. This toxic metalloid negatively impacts the physiology of a variety of organisms that ingest or filter contaminated food and water. Organisms with highly permeable skin are also prone to suffer with percutaneous absorption of arsenic^2^. In recent decades, human activities have accelerated the exposure of a variety of organisms to arsenic by the indiscriminate use of insecticides, herbicides, and preservatives that contain arsenic^3^. In some regions, arsenic contamination in the environment also occurs through natural routes^4^, arising from the erosive effects of the earth's crust^4,5^, and consequently, the leaching of this metalloid into water bodies. In these regions, anthropogenic interference such as habitat change and mining activities may dramatically increase arsenic concentrations in the environment, causing detrimental impacts on both wildlife and human health^6^.

Arsenic is present in different forms depending on its oxidation state (−3, 0, + 3, and + 5), being classified as both organic or inorganic, and its prevalence depends on pH and redox potential (Eh)^7^. Inorganic arsenic trioxide, or arsenite (As^3+^), is considered the most toxic form of arsenic because it reacts with the sulfhydryl groups of some proteins and enzymes, preventing their normal activity^8^. For instance, As^3+^ reduces functionality of antioxidant enzymes of the glutathione class that protect cells from damage caused by reactive species (RS), and pyruvate dehydrogenase that converts pyruvate into acetyl CoA^9^. Although many studies reported significant damage to cells resulting from exposure to As^3+^, studies have also shown that some organisms could turn on adaptive tolerance mechanisms as a response to As^3+^ exposure. For example, plants thriving in As^3+^ contaminated environments often show diverse physiological and cellular mechanisms of tolerance that are associated with hyperaccumulation of As^3+^^10–12^. Studies of arsenic tolerance in vertebrates have also revealed very complex routes of excretion mechanisms. As an example, Schlebusch and colleagues demonstrated that a human population inhabiting a naturally arsenic-contaminated region in the Andes of Argentina has evolved a novel excretion metabolism of arsenic, likely resulting from a single nucleotide mutation (SNP) in the AS3MT gene^13^. The AS3MT gene homolog in prokaryotes (*arsM*) has also been reported to be horizontally transferred between species^14^. Additionally, specialized proteins are able to chelate arsenic in some species of fish, thus reducing tissue damage when fish are exposed to different gradients of this metalloid^15^, as well as activating a myriad of other detoxification mechanisms^16^.

In amphibians, the relationship between environmental contamination and tissue damage is even more evident because amphibians rely on their skin for gas exchange and osmoregulation. Together with filter-feeder mollusks and crustaceans^17,18^, amphibians are often considered sentinels of ecosystem health because of their highly permeable skin to environmental contamination both in aquatic and terrestrial habitats^19,20^. The persistence of some amphibian populations in heavily contaminated environments challenges this assumption^21^, and offers an opportunity to study rapid vertebrate adaptation to harmful contaminants. Recent studies have shown that some amphibian species are highly adapted to high environmental concentrations of arsenic, whether recreated in the laboratory experiments or under natural field conditions^22^. Such adaptations involve amphibian metabolization or excretion of arsenic under different compositions, especially methylated organoarsenic compounds, mono, and dimethylarsonic acid^23^.

Microorganisms such as bacteria are also known to metabolize arsenic through several routes^24^, and it has been increasingly recognized that amphibians rely on skin-associated microorganisms (hereafter microbiota) for homeostasis and to fend off pathogenic infections^25^. Amphibians recruit their microbiota from the environment^26^, and our previous studies indicated that culturable microorganisms isolated from highly terrestrial Neotropical amphibian species are less tolerant to arsenic in challenge assays than the microbiota isolated from species that spend long periods of time in arsenic-contaminated water bodies^27^. Proença and colleagues also corroborated these results in a study that included amphibian species from Europe^28^. Thus, alongside host-driven responses to environmental contaminants, the composition of environmental and host-associated microbiota may help elucidate why certain amphibian species exhibit characteristics more akin to 'miners' rather than 'canaries in a coal mine'^21^. In line with the adaptive microbiome principle^29^, arsenic-mediated selection of microbial communities could be leading amphibians to recruit a protective biofilm layer enriched with arsenic-tolerant microorganisms, but this hypothesis remains to be tested.

Here, tested whether the amphibian skin-associated microbiota may confer adaptive tolerance to amphibians in regions heavily contaminated with arsenic. Using culture-based challenge assays, we (i) tested whether amphibians thriving at an arsenic contaminated region had a higher proportion of arsenic tolerant skin-associated bacteria than amphibians sampled at an arsenic-free region. We followed this observational study with an experiment in the laboratory where we (ii) tested whether amphibian-skin bacteria isolated at our arsenic contaminated region were the most efficient at reducing skin permeability to arsenic. This two-tiered approach allowed us to describe a pattern of environmental selection of arsenic-tolerant skin bacteria capable of protecting amphibians from intoxication, which helps explain the persistence of amphibian populations in water bodies heavily contaminated with arsenic. Our study highlights the potential role of evolutionary and metabolic processes in shaping the relationship between skin-associated microbiota and the physiological adaptations of vertebrates to challenging environmental conditions.

## Results

**Figure 1:**
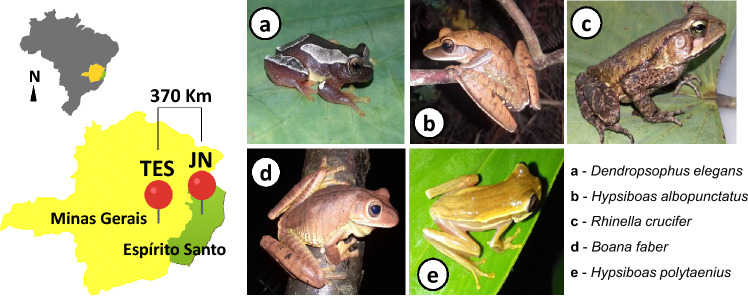
Sampling regions of our focal study species. *TES *Tripuí Ecological Station, an area naturally contaminated by metals, located in the state of Minas Gerais (yellow). *JN* municipality of João Neiva, uncontaminated area, located in the state of Espírito Santo (green). Images (**a–e**) highlight the morphological characteristics of the five species sampled in both regions.

We obtained a total of 644 bacterial isolates from our five focal amphibian species (Fig. 1); 337 from amphibians sampled at TES and 307 from amphibian sampled at JN (Table 1). The number of isolates obtained from the focal species in the two regions was very similar, except for *Hypsiboas albopunctatus* (with the highest number of isolates in contaminated area) and *Hypsiboas polytaenius* (with the highest number of isolates in uncontaminated area) (Table 1). Please see supplementary Table 1 for raw data.

**Table 1:** General features of the species captured in the two study areas.

	Calling sites	Reproductive mode	Skin	*n* specimens		*n* isolates	
				TES	JN	TES	JN
*Boana faber*	Inside nests built around the pond	Eggs placed directly in water	Smooth dorsal skin and slightly grainy ventral skin	3	3	60	64
*Hypsiboas albopunctatus*	Around the edges of puddles and ponds that contain emergent, floating or herbaceous vegetation			3	3	86*	43
*Dendropsophus elegans*			Smooth dorsal and ventral skin	3	3	58	48
*Hypsiboas polytaenius*	Tree trunks and leaves around the pond			3	3	39	69*
*Rhinella crucifer*	Around the pond		Dry and grainy skin on the belly and back	3	3	94	85
Total				15	15	337	307

*TES* Tripuí Ecological Station (contaminated area), *JN* João Neiva (non-contaminated area).

*p < 0.05.

**Figure 2:**
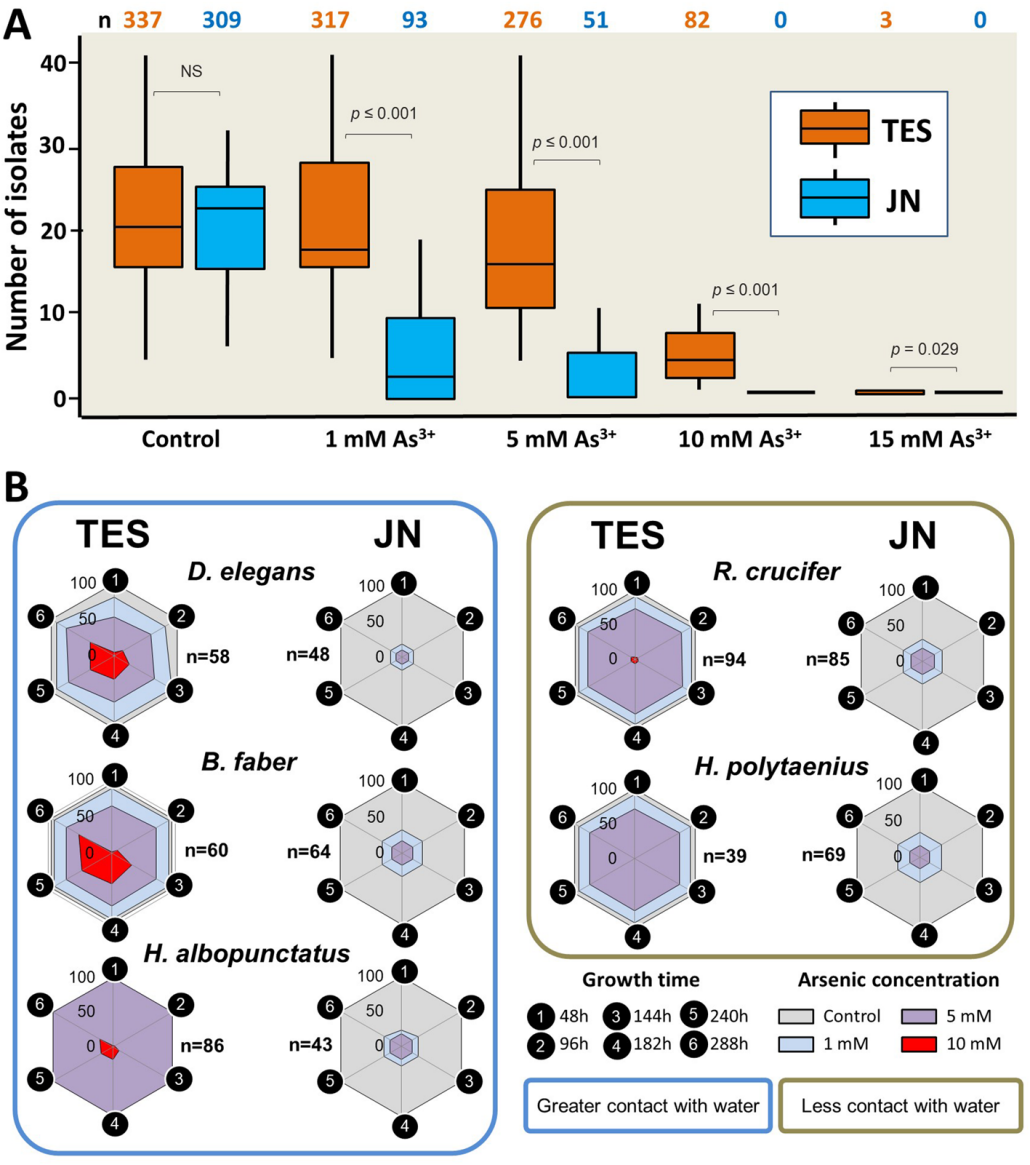
Analysis of the tolerance of bacterial isolates to different concentrations of arsenic. (**A**) Evaluation of the degree of tolerance of bacterial isolates in different arsenic concentrations. n: total number of tolerant isolates in the investigated conditions. TES contaminated area and JN uncontaminated area. n: total number of tolerant isolates in the investigated conditions. p-values of pairwise comparisons are shown. In the box plots, the median is depicted by a horizontal line, while the box marks the first and third quartiles. Vertical lines extend to the maximum and minimum values. (**B**) Evaluation of the degree of tolerance of bacterial isolates in our focal amphibian species sampled JN and TES sites. Numbers ranging from 0 to 100 represent the percentage of tolerance assessed at each of the six growth times. Colors represent arsenic concentrations to which isolates exhibited tolerance.

At lower arsenic concentrations (1 and 5 mM As^3+^), we found a significantly higher number of bacterial isolates tolerant to As^3+^ in species sampled at our arsenic contaminated site (TES) than from species sampled at our arsenic free site (JN) (whole model test: *x*^2^ = 279.949, d.f. = 7, P < 0.0001; Table 2; Fig. 2A). Strikingly, we found that the only bacterial isolates tolerant to high arsenic concentrations (10 mM and 20 mM As^3+^) in our challenge assay were isolated from amphibians sampled at our arsenic contaminated site (TES); none of the JN isolates tolerated high concentrations of As^3+^. Bacterial isolated from TES amphibians consistently showed greater arsenic tolerance than those isolated from JN frogs across all five amphibian species analyzed, although the magnitude of this difference varied among amphibian species. In addition, this difference in tolerance decreased at higher arsenic concentrations (see Table 1 for significant interactive term). We observed bacterial growth from TES isolates as early as 48 h after exposure to As^3+^ (Fig. 2B), although some of these isolates showed positive growth in the presence of As^3+^ only after over a week (240 to 288 h). Most bacterial isolates tolerant to 10 and 15 mM of As^3+^ were cultured from the following aquatic-breeding amphibian species in which we found a significantly higher number of bacterial isolates tolerant to As^3+^ in our focal TES region, *D. elegans,* (*t* = 2.974, *P* = 0.006), *H. albopunctatus* (*t* = 2.459, *P* = 0.020), *and B. faber* (*t* = 1.675, *P* = 0.105), in contrast to our two focal species with the highest levels of terrestriality or arboreality (*H. polytaenius: t* = 0.083, *P* = 0.934; *R. crucifer: t* = 1.728, *P* = 0.095; Fig. 2B).

**Table 2:** General linear model (GLM) with Poisson distribution and log link for number of bacterial isolates as the single response variable.

Variable	d.f	*x*^2^	*P*
As^3+^ concentration (mM)	1	202.452	< 0.0001
Region	1	104.566	< 0.0001
Region × As^3+^ concentration (mM)	1	45.826	< 0.0001
Amphibian species	4	15.550	0.004

The following variables were included as explanatory factors: amphibian species, As^3+^ concentration (0 "control", 1, 5, 10, and 15 mM/ml), region (JN or TES), and the one-level interaction.

**Figure 3:**
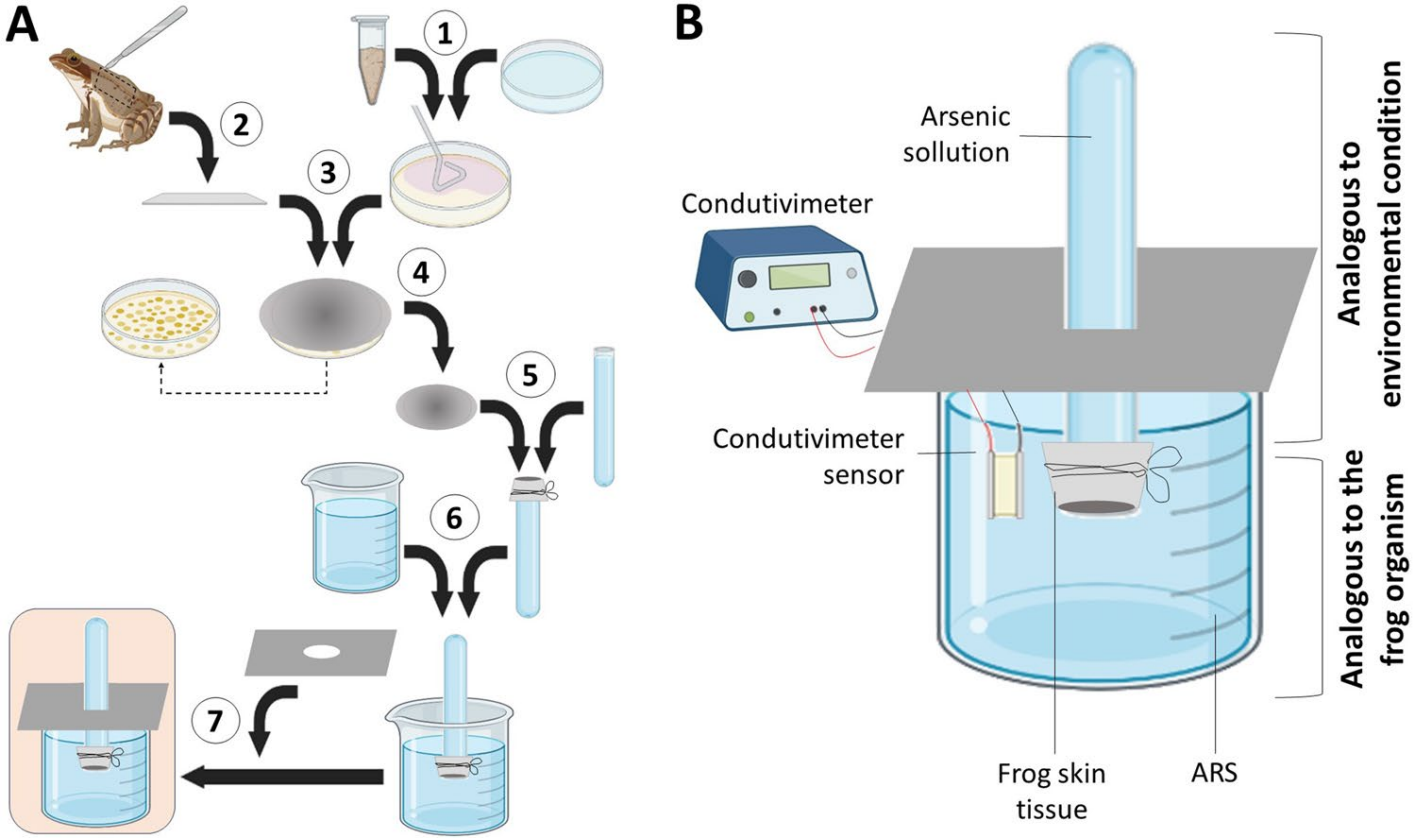
Steps involved in the construction of the apparatus for simulating exposure to arsenic and analysis of the influence of skin-associated microbiota on the permeability of this contaminant. (**A**) Ilustrative summary of the steps described in the methodology. Numbers 1 to 7 represent the following steps: 1—reactivation of the selected bacteria; 2—in parallel, fragments of bullfrog skin (*Lithobates catesbeianus*) were prepared; 3—skin fragments were placed in contact with bacterial cultures in Petri dishes and incubated at 28 °C for 24 h; 4—after incubation, skin fragments were removed from the Petri dish and separated; 5—skin fragments were tied to the open end of the test tube containing the arsenic solution (5 mM NaAsO_2_ final concentration); 6—test tube containing the tied skin was exposed to Amphibian Ringer's solution (ARS); 7—test tube was suspended by a rigid black paper plate, thus limiting the contact of the ARS with the environment. (**B**) Method for measuring the change in conductivity due to the permeabilization of arsenic through the amphibian skin, previously treated with bacteria.

To verify the influence of bacteria on the permeability profile of anuran skin to arsenic in solution, we developed a novel low-cost methodology (Fig. 3).

**Figure 4:**
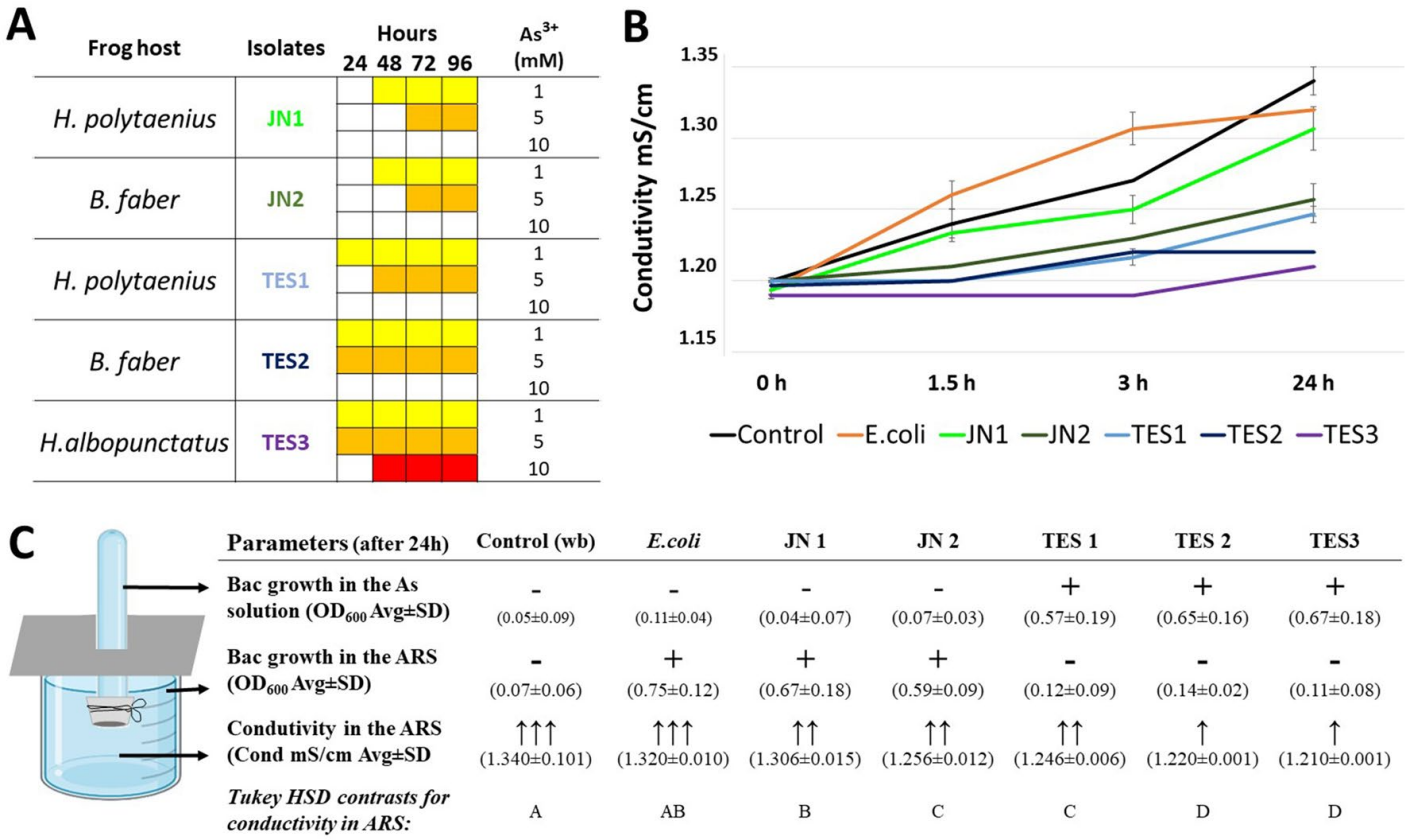
Qualitative analysis of the results obtained from the amphibian simulation apparatus to contaminated environments. (**A**) Growth chronology and tolerance profile of selected isolates at different arsenic concentrations. JN1 and JN2: isolates obtained from an uncontaminated area. TES1, TES2, and TES3: isolates obtained from contaminated area. Colored squares represent the times in which the growth rate of bacterial isolates was confirmed in the following arsenic concentrations (1 mM yellow, 5 mM orange, and 10 mM red). (**B**) Change of conductivity in Amphibian Ringer's solution after 24 h. The observed increase in conductivity was directly associated with diffusion of arsenic through the amphibian skin. Error bars (SE) were determined from three independent experimental trials. (**C**) Summary of qualitative results associated with the use of the apparatus. + represents positive bacterial growth and- negative. Values within parentheses are average ± standard deviation. Upward facing arrows indicate increased conductivity after 24 h of testing. Levels not connected by the same letter are significantly different according to aposteriori Tukey HSD test for multiple comparisons. The number of arrows is directly associated with increases of conductivity values.

The selection of isolates used in the permeability test for this metalloid was defined based on the potential for arsenite tolerance (Fig. 4A). Our results pointed to significant differences in skin permeability when utilizing bacterial isolates from our focal areas with distinct arsenite contamination (F = 108.886, Df = 6, P < 0.0001; Fig. 4B). Bullfrog tissue exposed to JN1 bacterial isolate showed similar arsenite conductivity to tissue exposed to *E. coli*, and only slightly greater conductivity of tissue fragments without associated bacteria (i.e., ARS control). Arsenite conductivity of tissue fragments exposed to bacterial isolates JN2 and TES1 were slightly lower than those exposed to bacterial isolate JN1, but higher than conductivity observed in skin treated with TES2 and TES3 isolates (Fig. 4B). We found that bacterial growth was only observed in samples of ARS exposed to control skin or skin treated with JN isolates. In contrast, skin fragments treated with TES isolates only grew on frog skin exposed to As^3+^ solution (Fig. 4C). In contrast, skin fragments treated with bacteria isolates obtained at the arsenic contaminated area only grew in the system containing arsenite solution (Fig. 4C).

## Discussion

Physiological adaptations have been gaining prominence as some of the most significant factors related to amphibian adaptations in environments with high selective pressure, such as areas naturally contaminated by heavy metals and metalloids. Moryart and colleagues described, for example, the ability of the American amphibians *Rana clamitans* and *Anaxyrus americanus*, collected from a site with elevated arsenic concentrations in Nova Scotia, Canada, to transform this metalloid from its highly harmful inorganic state to a less damaging organic state^23^, thereby reducing cellular and metabolic damage. Apart from physiological adaptations, numerous studies have been conducted to comprehend the diversity and potential interactions of the microbiota associated with amphibians in host adaptive processes^29,30^. As a result, research on the adaptive nature of host microbiomes has become important for animal conservation. Most of this new wave of research has relied on metagenomic approaches, with a focus on the study of microbiome dysbiosis induced by the presence of the emerging amphibian chytrid fungus *Batrachochytrium dendrobatidis*^31–35^. Some studies revealed that levels of bacterial diversity vary greatly among species, and variation in microbial community diversity appears to regulate the structure of bacterial communities in amphibians^36^. The skin-associated bacteria of amphibians are acknowledged for their defensive role against pathogens or environmental pollutants^26,37^. However, their ecological interactions and the complexity of functional traits are not yet fully understood.

Despite the increased focus on studies centered around the amphibian skin microbiome, there is still a notable lack of culture-based research investigating the role of host-associated microorganisms in enhancing host tolerance to environmental contaminants. In a recent effort to address this knowledge gap, Assis and colleagues examined 221 bacterial isolates from 188 individual frogs belonging to four amphibian species and their findings suggest that the environment plays an important role in modifying the microbiota^38^. Similarly, a study carried out with anurans from the IQ region (naturally contaminated by metals and metalloids)^39^ found that animals that had regular contact with waterbodies were found to have microbiota that was more tolerant to arsenic when compared to those that only partially depended on water to reproduce^27^. Furthermore, the microbiota associated with amphibian species that use water bodies year round showed an enhanced ability to produce biofilms^27^. The production of biofilms could potentially aid in the persistence of microorganisms in animal tissue and provide enhanced protection against contaminants that arise due to physicochemical fluctuations in the external environment^27^. Proença and colleagues corroborated some of these results in a study focusing on culturable microbiota of Perez frogs (*Pelophylax perezi*) collected in regions contaminated and uncontaminated with metals^28^. The study showed that the culturable microbiota, while not completely representative of the bacterial diversity in the environment, can provide valuable insights into the ecology and processes involved in bacterial-animal interactions.

In our study, arsenic was chosen as a reference due to its high toxicity to living organisms^1^ and its reported abundance in the IQ region^40–42^. We found that bacterial isolates from amphibians sampled at our focal arsenic contaminated area were much more tolerant to As^3+^ than those from species sampled at an uncontaminated area. Furthermore, we did not detect significant differences in the total number of bacterial isolates from the amphibian species sampled in both regions, further supporting our results. The results indicate that the presence of arsenic may have played an important role in shaping the host-associate microbiota, as previous studies have shown that environmental factors can significantly impact the composition and diversity of both host-associated and environmental microbiota.^38,43^. Consistent with our hypotheses, bacterial isolates from *Boana faber*, *Dendropsophus elegans*, and *Hypsiboas albopunctatus*, amphibians with a more aquatic lifestyle, exhibited higher tolerance to the highest concentration of As3 + (10 mM), compared to those from *Hypsiboas polytaenius* and *Rhinella crucifer*, whose life cycles are less water-dependent^44^. These data reinforce the results previously published by our team^27^, highlighting that the most water-dependent amphibian species were those with the highest percentage of bacterial isolates with high tolerance to metals. Both studies demonstrated that habitat use could also influence the composition of the host-associated microbiota, reiterating the role of microorganisms in the adaptation of amphibians to arsenic contaminated sites^23^.

Our skin permeability experiment revealed that bacterial isolates obtained from species sampled in an uncontaminated area (isolates JN1 and JN2) and with lower arsenic tolerance had a higher rate of arsenic migration from the metalloid solution (simulating the environment) to the ARS (simulating the anuran fluids) that had to diffuse through the epithelial tissue. Such diffusion induces a change in conductivity in the ARS solution because the electrical conductivity is affected by the concentration of ions in the solution^45^. These results differ from those obtained using bacterial isolates from amphibian species sampled at our arsenic-contaminated site, which are presumably more arsenic-tolerant. For isolates TES2 and TES3, the observed change in conductivity through the ARS solution was much smaller, which supports the tolerance profile of these isolates to As3 + and, therefore, the increased protective function for amphibians. A curious fact observed for isolates with greater protective power is that they showed a positive growth rate in the contaminant solution, which could suggest a chemoautotrophic metabolic profile, whereas isolates with lower protection potential showed microbial growth only in ARS. It is important to highlight that our apparatus developed to verify permeability tests on the epithelial tissue of frogs could be further improved in future experiments. Replacing frozen skin with fresh samples, examining the structure of the epithelial tissue before and after experiments, and developing techniques to measure real-time changes in media (such as arsenic or Ringer's solutions) could be implemented.

**Figure 5:**
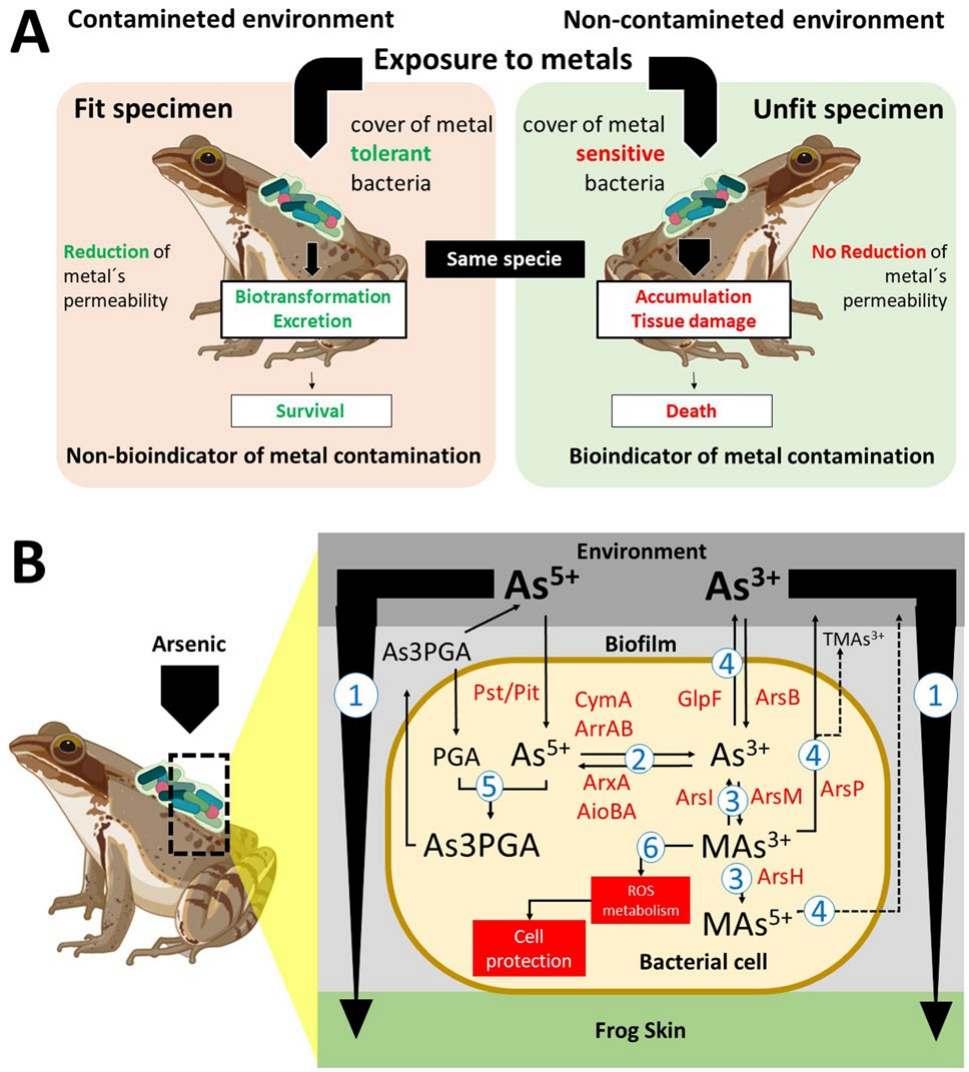
Model proposed to suggest how the epithelial microbiota interferes in the adaptation of the anurans in contaminated environments. (**A**) Resilience (left) and animal susceptibility are dependent on the tolerance profile of the associated bacterial species. Amphibian species that have tolerant microbiota may not be good indicators of environmental contamination. (**B**) Metabolic diversity is found in bacteria associated with arsenic metabolism. 1—Biofilm reduces metal contact with animal tissue; 2—redox reactions reduce ion toxicity; 3—biotransformation reactions transform more toxic species into less toxic ones; 4—efflux of ions or molecules carrying the contaminant; 5—chelation of harmful ions to organic molecules; 6—activation of oxidative species metabolism, increasing cell protection.

Taken together, these data indicate that host-associated bacteria may serve as the first line of defense to protect the epithelial tissue, allowing amphibians to survive and adapt to naturally contaminated environments. Our findings suggest that the resilience of amphibians to environmental contamination may be closely linked to the metabolic and functional capacity of their epithelial microbiota^46,47^ (Fig. 5A). This, in turn, could contribute to the persistence of amphibian populations and the structure of their communities^37,48^.

Although our research has focused on a singular detoxification pathway, it is important to note that host-associated bacteria could detoxify metals and metalloids through several mechanisms (as illustrated in Fig. 5B). Of these mechanisms, the capacity to produce biofilms is particularly noteworthy as it reduces the diffusion and direct contact of the metal with animal tissue. Other mechanisms involve the ability to promote redox processes, which can convert toxic ions into less harmful ones, the potential for biotransformation (leading to the formation of organometallic complexes), the efflux of contaminants out of the cell, the chelation of ions by organic molecules, and the increase in the metabolic profile of oxidative stress-associated systems^49^. This context emphasizes that amphibians thriving in heavily polluted areas serve as excellent models for studying animal-microbiota interactions, with contaminated areas serving as hotspots for comprehending the evolutionary profiles of host-associated microbiomes.

## Conclusions

Our study indicates that the tolerance of skin-associated microbiota of amphibians from arsenic-contaminated areas might be crucial to the adaptation of amphibian populations, leading to a decrease in tissue permeability to As^3+^. We identified a pattern of environmental selection of arsenic-tolerant skin-associated bacteria that can protect amphibians from intoxication, thus explaining the persistence of amphibian populations in water bodies heavily contaminated with arsenic. As a result, the term "bioindicator" widely used for amphibians, should be used with caution. Amphibian species found in naturally contaminated environments have associated skin microbial taxa that can mitigate the harmful effects of arsenic contamination, in addition to physiological adaptations. In agreement with our predictions (see Fig. 5), our findings underscores how evolutionary and metabolic processes may influence the interplay between skin-associated microbiota and the physiological adaptations of vertebrates to demanding environmental conditions.

## Methods

### Study sites and species

We sampled amphibians in two regions with contrasting levels of arsenic contamination: Estação Ecológica do Tripuí (hereafter TES), an area heavily contaminated with arsenic and other heavy metals (As(V)—9 to 224 μg∙L^−1^^50^, and João Neiva (hereafter JN), an area without known arsenic contamination. TES is located in the municipality of Ouro Preto, State of Minas Gerais, whereas JN is located ~ 370 km east of Ouro Preto in the municipality of João Neiva, State of Espírito Santo (Fig. 1). TES is located at a transition region between two Brazilian ecoregions listed by the IUCN as global biodiversity hotspots: the Atlantic Forest and the Brazilian Savanah (Cerrado). Located in the metallurgical zone of Ouro Preto (20°23′45″ S e 43°34′33″ W), in the Tripuí Creek sub-basin, TES has an area of 3.37 km^2^ and an has elevation ranging between 1280 and 1450 m^51^. The climate at TES is subtropical, with temperate summers and dry winters, annual rainfall of ~ 1600 mm, and an average annual temperature of 18 °C^52^. Sampling sites within TES were located around Lago Fortes along a ~ 300 m stretch of Ribeirão Tripuí, an area previously sampled by Cordeiro et al.^27^ and with known high levels of heavy metals and metalloids^40–42^. Our focal sampling region without arsenic contamination (JN) consists of an agricultural landscape in the municipality of João Neiva. The municipality has an area of 272.30 km^2^ and is located between latitudes 19°37′20″ and 19°47′57″ and longitudes 40°31′37″ and 40°20′28″ W within the Atlantic Forest ecoregion^53,54^. The climate of JN is monsoon-influenced humid subtropical, characterized by dry winters and rainy summers, monthly rainfall of ~ 80 mm, and an average annual temperature between 22 and 32 °C^52^.

We sampled three male individuals of each of the following five focal amphibian species at both of our sampling regions: *Boana faber, Dendropsophus elegans, Hypsiboas albopunctatus* (*three highly aquatic amphibian species*)*, and Hypsiboas polytaenius,* and *Rhinella crucifer* (*two species less dependent on water bodies throughout their life cycle*). Samples took place between February and September, and life-history characterizations for each species are summarized in Table 1. Frogs were captured using an active night search. At both locations, we tagged captured individuals using a visible implant fluorescent elastomer (VIFE) to avoid recaptures and unwanted pseudoreplication in our analyses^55^.

### Sample collection, bacterial isolation, culturing and preservation

Immediately after capture, we washed animals with two baths of autoclaved distilled water. We then sampled skin of individual amphibians with a sterile swab (Olen Ref K41-0101C) according to protocols described by Hyatt et al.^56^. We used disposable sterile materials for all procedures according to Cordeiro et al.^27^. All animals were released at their capture location immediately after swabbing. We individually stored skin swab samples in sterile 15 mL Falcon tubes containing 3 mL of liquid Luria–Bertani (LB) medium^57^. We decided to use the nutrient-rich LB medium because it provides a broad base of nutrients that fosters the growth of a large number of bacterial taxa. We transferred 1 mL of each stock sample to a sterile 1.5 mL centrifuge tube. We established progressive 1/10 dilutions up to the 10^8^ dilution factor containing bacterial inoculum. We then inoculated 100 µL of the bacterial solution at the 10^8^ dilution into individual Petri dishes containing LB medium with pH adjusted to 7.0, plus 2 mg/L of Viper 700^®^ (methyl thiophanate) using a Drigalski loop, keeping Petris at 28 °C for 48 h. After this period, samples of all bacterial colonies that grew on Petris were replated with the same medium, incubated for another 48 h at 28 °C, and then preserved. For preservation, we transferred each isolate to a new 1.5 mL tube containing 1.0 mL of liquid LB medium. We then incubated these tubes for 48 h at 28 °C. After this period, we added glycerol to each tube at a final concentration of 30% (v/v). We supplemented our bacterial database for two (*Boana faber* and *Rhinella crucifer*) of our five focal amphibian hosts with samples previously collected at TES by Cordeiro and collaborators^27^.

### Arsenite resistance assay

To analyze the resistance of each bacterial isolate to arsenic we used cultures growing in Petri dishes with solid LB medium at different concentrations of As^3+^ (1 mM, 5 mM, 10 mM, 15 mM, and 20 mM), in addition to a control dish containing only solid LB medium. We transferred bacterial isolates to 96-well plates using a replicator. We then transferred these samples to Petri dishes with different concentrations of As^3+^ and incubated them at 28 °C using a 96-tip multi-replicator. We photographed each culture at 48 h intervals over 12 days to monitor the growth of bacterial isolates. We quantified As^3+^ tolerance by measuring colony growth over time at each As^3+^ concentration.

Although unlikely, it should be noted that visible growth of bacteria on As^3+^-containing media after 48 h might not necessarily indicate bacteria that were resistant or tolerant to arsenic at the time of isolation. These observed colonies could potentially be mutants that emerged early in the growth assay and subsequently replicated to the point of visibility by the end of the growth period. In such a scenario, visible growth may not accurately reflect resistance of bacteria collected from the skin, but rather indicate bacteria with higher mutation rates or potential variations in RNA polymerase activity. However, it is more plausible that these bacterial isolates were indeed resistance or tolerance to As^3+^ at the time of isolation, and exhibit slow growth rates.

### Skin frog permeability apparatus development

To test whether isolated skin-bacteria could reduce the permeability of amphibian skin to arsenic, we developed an ecologically realistic apparatus that measures skin permeability to As^3+^. The building of this apparatus involved assembling test tubes, beakers, and a conductivity meter with the aid of rubber bands, and by adding solutions mimicking the animal's bodily fluids and the environment contaminated by arsenic; a detailed representation is sequentially depicted in Fig. 3.

Initially, we aseptically excised fragments of bullfrog skin (*Lithobates catesbeianus*) from a frog farm/slaughterhouse donation and keep individually wrapped fragments frozen until the onset of the experiment. Prior to the experiment, we thawed skin at room temperature and cut it into square-shaped fragments with 6 cm edges. These fragments then went through a disinfection phase, remaining immersed in a 0.01% sodium hypochlorite solution for 3 min, followed by two washing steps with ddH20, immersing them for 3 min in each step. In parallel, the bacterial isolates selected for this assay were pre-inoculated in LB medium at 28 °C under agitation of 250 rpm in a shaker, and 25μL were then transferred and spread in LB agar medium in the Petri dish at the moment in which the growth solution achieved an optical density (OD600nm) of 0.6. Finally, we exposed the outer epidermal layer of each skin fragment to bacterial cultures in Petri dishes, and incubated each dish at 28 °C for 24 h.

After this period, we removed the skin from each Petri in laminar flow hood and mounted individual skin fragments to the mouth of test tubes containing an arsenic solution of 5 mM NaAsO_2,_ avoiding contact with the bacterial growth zone as much as possible. We mounted the epidermal layer, which was previously colonized by our focal bacteria, towards the inside of each tube containing As^3+^ solution. We then turned tubes upside down into a container with amphibian Ringer's solution (ARS—6.6 g of NaCl, 0.15 g of KCl, 0.15 g of CaCl_2_, and 0.2 g of NaHCO_3_ dissolved in 1 L of distilled water^58^), which simulated osmotically realistic amphibian body fluids. To secure the test tube while maintaining the inner surface of the skin in continuous contact with ARS (suspended 2 cm from the bottom of the beaker), we used a rigid piece of black paper fitted to the center of the test tube and supporting the weight of the tube through the edges of the beaker, thus limiting the contact of ARS with the outer environment. The sensor of a conductivity meter was inserted into the ARS.

### Arsenic frog skin permeability test

To evaluate the apparatus, we selected five (out of 644) bacterial isolates for these assays: two bacterial isolates from JN tolerant to 1 (JN1 from *H. polytaenius*) and 5 mM (JN2 from and *B. faber*) of As^3+^, and three isolates from TES tolerant to 5 (TES1 from *H. polytaenius*), 10 (TES2 from and *B. faber*), and 15 (TES3 from *H. albopunctatus*) mM of As^3+^. Additionally, we included the *E. coli* strain DH10B (susceptible in the presence of arsenic) in the trial as a control. We reactivated each of these six bacterial isolates and grew them on solid LB medium (on 60 × 15 mm Petri dishes) for two days at 28 °C. We measure conductivity by placing the conductivity meter probe in contact with ARS at 0, 1.5, 3, and 24 h after the onset of the experiment, taking conductivity readings in triplicate. At the end of this experiment, we also measured and compared bacterial growth rates among isolates growing (based on optical density—OD and turbidity) in in both ARS (mimicking amphibian body fluids) and As^3+^ solution (mimicking the outside environment).

### Indirect evaluation of bacterial growth profile in the apparatus

In addition to conductivity, we also quantified changes in turbidity (a metric of bacterial growth) on both arsenic solution and ARS. We validated our interpretations through reactivation of bacteria from both solutions in culture medium. We reactivated bacteria by inoculating and spreading 10 μL of each solution with a Drigalski loop in individual Petri dishes containing LB medium, with pH adjusted to 7.0, and kept at 28 °C for 48 h. To determine the absorbance, three samples of each culture (ARS and As^3+^ solution) were taken (1 ml) and read in a spectrophotometer (Palo Alto. CA, USA) in visible light with a wavelength of 600 nm.

### Statistical analysis

To compare bacterial growth on arsenic medium between isolates from TES and JN amphibians, we performed a generalized linear model (GLM) with a Poisson distribution and a log link^59^. In this GLM, bacterial count was treated as the response variable and the isolation region (JN or TES), the concentration of arsenic and the interaction between them were included as the explanatory variables. In addition, we included amphibian species as an additional fixed effect in the model. We also used radar plots to visualize temporal data of bacterial growth time that was not included in our GLMs.

For the conductivity and bacterial growth assays measured by absorbance, average and standard deviation of the experimental replicates were calculated. We also performed a GLM with normal distribution and identity link to test the how conductivity values measured at 24 h (continuous response variable) varied among five bacterial isolates grown from our two regions vs. *E. coli* vs. control group. We followed this GLM with a Tukey test for pairwise multiple comparisons. All statistical analyses were performed using R (v. 4.0.4)^60^.

### Ethics statement

Sampling and handling permits were provided by ICMBio SISBIO #29.219-4, IEF #UC 087/13, and CEUA 138/2013.

### Supplementary Information

Supplementary Figure 1.

Supplementary Table 1.

## Data Availability

All raw data were deposited at OSFRegistries public repository (https://osf.io/) with doi 10.17605/OSF.IO/9TNRC (https://osf.io/9tnrc/).
