# Supplementary figures and images for: Amphibian tolerance to arsenic: microbiome-mediated insights
Source: Sci Rep. 2024 May 3;14:10193. doi: 10.1038/s41598-024-60879-w (PMC11068734; doi:10.1038/s41598-024-60879-w)

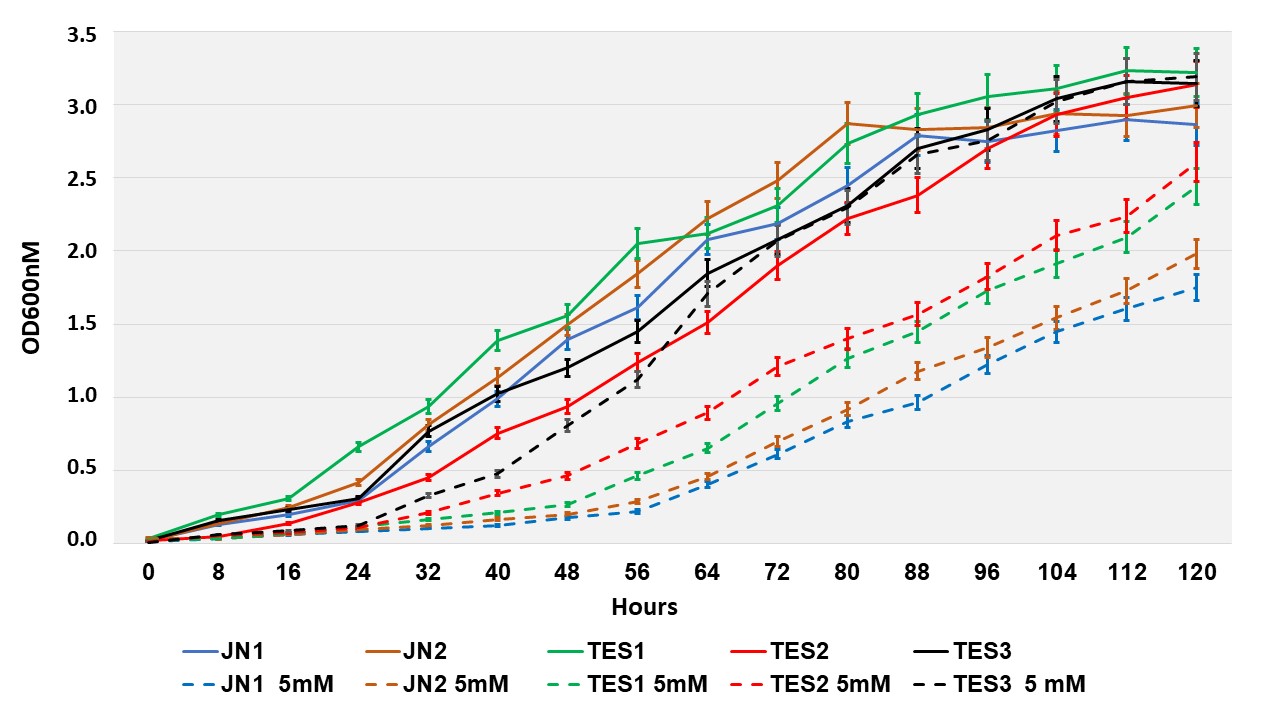

Supplement: Supplementary file 1 — Supplementary Figure 1. [file 41598_2024_60879_MOESM1_ESM.jpg]
